# Characteristic Evaluation of Gas Chromatography with Different Detectors for Accurate Determination of Sulfur Hexafluoride

**DOI:** 10.3390/molecules29040787

**Published:** 2024-02-08

**Authors:** Susu Pan, Tiqiang Zhang, Guocheng Zhang, Zhenqi Yang, Duan Feng, Zhikuan Zhou, Xuelei Ning

**Affiliations:** 1 Division of Ecology Environment and Energy Resources, Beijing Institute of Metrology, Beijing 100012, China; panss@bjjl.cn (S.P.); yangzq@bjjl.cn (Z.Y.); fengduan@bjjl.cn (D.F.); zhouzq@bjjl.cn (Z.Z.); ningxl@bjjl.cn (X.N.); 2 Center for Environmental Metrology, National Institute of Metrology, Beijing 100029, China; zhangtq@nim.ac.cn

**Keywords:** sulfur hexafluoride, greenhouse gas, gas chromatography, certified reference material, calibration curve, analytical accuracy

## Abstract

Sulfur hexafluoride (SF_6_), which survives in the atmosphere for an extremely long period of time, is the most potent greenhouse gas regulated under the Kyoto Protocol. So, the accurate monitoring of atmospheric SF_6_ plays an important role in the study of the control policies for reducing greenhouse gas emissions. The instruments for SF_6_ measurement are typically calibrated using certified reference materials. The concentrations of the commercially available SF_6_ reference materials usually have a broad range, from 1 μmol/mol to 6000 μmol/mol. Some characteristics including sensitivity, linear range, relative standard deviation, and accuracy are crucial for the determination of SF_6_ in such a broad concentration range. Therefore, the selection of a proper detector for the accurate determination of SF_6_ with such a broad range is extremely important to establish a gas chromatography (GC) method for developing SF_6_ reference materials. In this paper, several typical GC methods with different detectors, including a thermal conductivity detector (TCD), a pulsed discharge helium ionization detector (PDHID), and a flame photometric detector (FPD), were carefully established for the accurate determination of SF_6_ with different concentrations. The results show that an FPD detector has a relatively narrow linearity range, thus a quadratic equation should be established for building a calibration curve. The PDHID and TCD have good linearity with coefficients of 1.0000 in the concentration range of 10–100 μmol/mol (using a PDHID), and 100–1000 μmol/mol (using a TCD), respectively. Further considering the measurement errors of indication results, the PDHID is suitable for SF_6_ measurement when the concentrations are below 100 μmol/mol, whereas the TCD is suitable for SF_6_ measurement when the concentrations are over 100 μmol/mol. These results provide useful guidance in choosing an appropriate GC detector for the accurate determination of SF_6_, which are especially very helpful for developing SF_6_ reference materials.

## 1. Introduction

Sulfur hexafluoride (SF_6_) is a colorless, odorless, non-toxic, non-combustible, and non-corrosive gas. Its highly stable and electronegative performance makes it both an ideal dielectric and insulator, which is widely used in electrical equipment [1,2,3,4], the magnesium and aluminum industries [5], semiconductor manufacture, meteorology, biotechnology, aerospace, refrigeration, laser, medicine, and other fields [6].

Of all the greenhouse gases regulated under the Kyoto Protocol [7], SF_6_ is the most potent greenhouse gas [8] and persists in the atmosphere for an extremely long time, roughly 800–3200 years [9,10,11].

The earliest measurements of SF_6_ reported a mole fraction of <1 pmol/mol (or ppt, parts per trillion) [12,13,14]. The mass spectrometer (MS) and electron capture detector (ECD) are most often employed as gas chromatographic detectors for measuring trace SF_6_ in the atmosphere. Most ambient SF_6_ measurements using ECD typically have a precision of 2%. Pre-concentration may provide more precise results, although it is less frequent than loop-injection air samples and comes with more complexity [11].

SF_6_ emissions from electrical equipment occur during production, routine maintenance, refill, leakage, and disposal [15,16]. Random failure or deliberate or accidental venting of equipment may also cause high levels of emissions. For instance, in a single incident in 2013, a broken seal resulted in the emission of 113 kg of SF_6_ [17]. Such gas leakage incidents could be prevented through using an alarm or other gas leakage detection system.

All the instruments for SF_6_ measurements are typically calibrated against certified reference materials (CRMs) [18,19,20,21,22,23]. The concentrations of commercially available SF_6_ reference materials usually have a broad range from 1 μmol/mol to 6000 μmol/mol. Choosing a proper detector for the accurate determination of SF_6_ is extremely important to establish a GC method for developing SF_6_ reference materials. In this study, typical GC methods with different detectors, including TCD, PDHID, and FPD were carefully researched and established for the accurate determination of SF_6_. The detectors’ performance, including precision and accuracy, is comprehensively discussed.

## 2. Results and Discussion

### 2.1. Determination of SF_6_ Using GC-FPD

**Figure 1:**
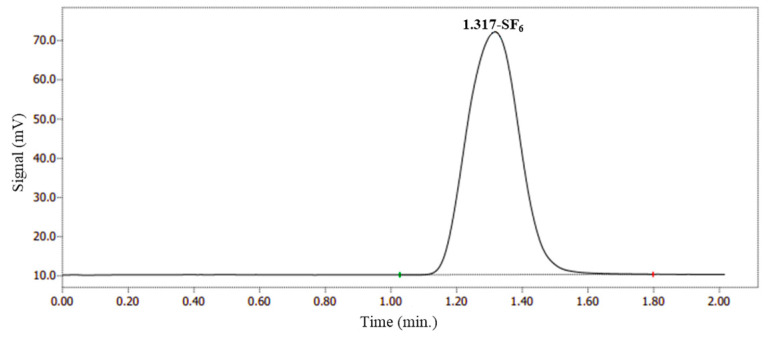
The chromatogram of SF_6_ CRM (10 μmol/mol) using FPD.

A typical chromatogram of SF_6_ measured using an FPD is shown in Figure 1. The main peak at 1.317 min corresponds to SF_6_. For repeatability, the RSD value of the peak area obtained from six standard injections of 10.0 μmol/mol was calculated to be 0.62%.

**Figure 2:**
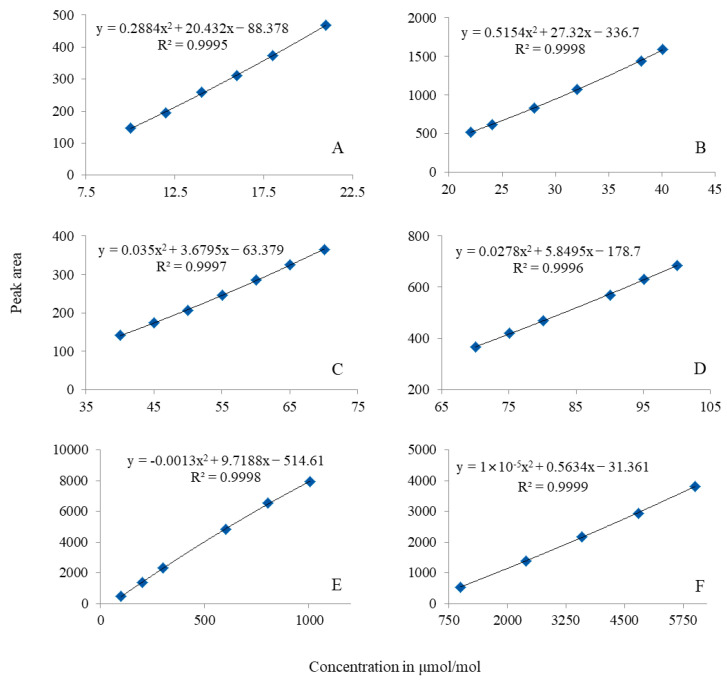
Calibration curves of SF_6_ in nitrogen using GC-FPD. (**A**): 10–20 μmol/mol; (**B**): 20–40 μmol/mol; (**C**): 40–70 μmol/mol; (**D**): 70–100 μmol/mol; (**E**): 100–1000 μmol/mol; (**F**): 1000–6000 μmol/mol.

The broad range from 10 μmol/mol to 6000 μmol/mol commonly exists for SF_6_ reference materials. Establishing a single equation describing the response of the FPD against SF_6_ concentrations in such a wide range is impossible. GC-FPD was unsuitable for the quantitative analysis of SF_6_ concentration if both the nonlinear response and the quenching effect were not considered [24], particularly for sulfur concentrations in a relatively wide linear range. If the calibration curves have a relatively narrow range, a quadratic equation, *y = a + bx + cx^2^*, can be used to establish a calibration equation. The peak area and SF_6_ concentrations were denoted as *y* and *x* in the calibration curves. Six narrow concentration ranges were divided among the wide concentration range. Thus, six calibration curves were constructed via plotting the peak areas against the concentrations, as shown in Figure 2. The coefficient of determination (*R*^2^), used to express linearity, was calculated to be ≥0.9995 for all equations.

The accuracy, reflecting the performance characteristics, of the calibration curve was also evaluated. The relative measurement error was used to evaluate the accuracy of the calibration curve. A sulfur hexafluoride in nitrogen CRM (GBW08124), with a certified value of 10.0 μmol/mol was chosen as an “unknown” and analyzed using GC-FPD under the same analytical conditions. The measurement value of this SF_6_ CRM was calculated using the established calibration equation: *y* = 0.2884*x*^2^ + 20.432*x* − 88.378. As a result, the measurement value was calculated to be 9.983 μmol/mol. The uncertainty of the measurement was evaluated to be 1.8% (*k* = 2). Equation (1) was used to evaluate the relative measurement error, where Δ is the relative measurement error; *x* is the measurement value based on the established equation; and $x_{s}$ is the certified value. Finally, the relative measurement error was calculated to be −0.17%, which is far smaller than the expanded uncertainty of the used CRM, which certified 1% (*k* = 2), and shows very good accuracy of GC-FPD through building up the equation for the narrow concentration range.

(1) $$\Delta =\frac{x-x_{s}}{x_{s}}\times 100\%$$

### 2.2. Determination of SF_6_ Using a PDHID

**Figure 3:**
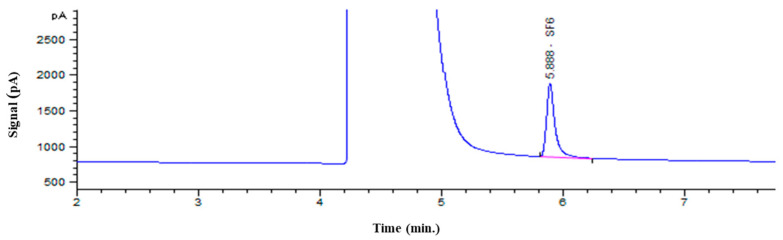
The chromatogram of an SF_6_ CRM (10 μmol/mol) using PDHID. The peak at 4.2~5.2 min and 5.888 min corresponds to N_2_ and SF_6_, respectively.

A typical chromatogram of an SF_6_ CRM using PDHID is shown in Figure 3. The peak at 5.888 min corresponds to SF_6_. For repeatability, the RSD value of the peak area obtained from six injections of 10 μmol/mol was calculated to be 0.18%.

**Figure 4:**
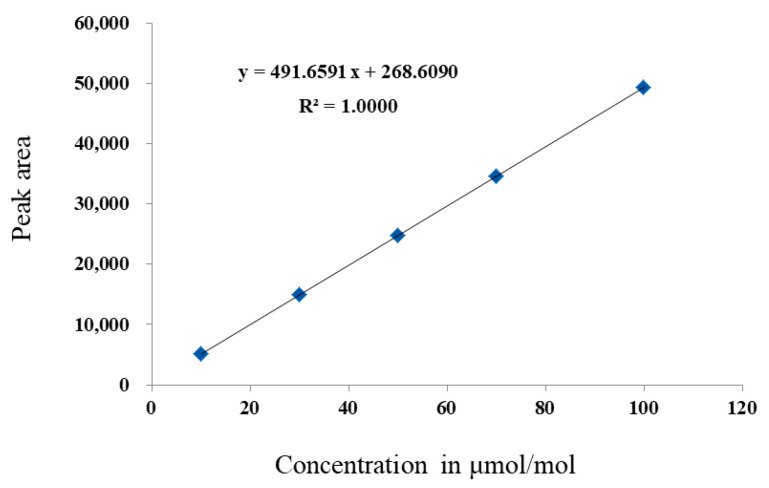
Calibration curves of SF_6_ in nitrogen using GC-PDHID, 10~100 μmol/mol.

The PDHID has a good linearity for the range of 10–100 μmol/mol, showing a determination coefficient (*R*^2^) of 1.0000 (Figure 4). The same SF_6_ CRM (GBW08124) with a certified value of 10.0 μmol/mol was chosen as an “unknown” and was analyzed using GC-PDHID. The measurement value of this SF_6_ CRM was calculated according to the established calibration equation: *y =* 491.6591 *x +* 268.6090. As a result, the measurement value was calculated to be 10.019 μmol/mol. The uncertainty of the measurement was evaluated to be 1.2% (*k* = 2). The relative measurement error was calculated to be 0.19%.

**Figure 5:**
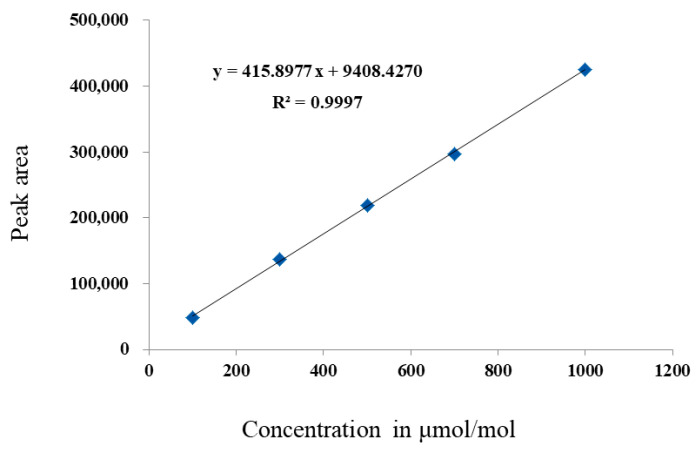
Calibration curves of SF_6_ in nitrogen using GC-PDHID, 100~1000 μmol/mol.

The extent of the calibration curve of SF_6_ studies on 100–1000 μmol/mol is shown in Figure 5. It was clear that the calibration was linear, as a result of a coefficient of determination (*R*^2^) of 0.9997. The linearity is not quite as good as that from the linear calibration range of 10–100 μmol/mol. To evaluate the accuracy for this calibration curve, another SF_6_ CRM (GBW08124) with a certified value of 100 μmol/mol was chosen as an “unknown” and was analyzed. The measurement value was calculated through using the established equation: *y* = 415.8977 *x* + 9408.4270. The measurement value was calculated to be 99.31 μmol/mol. The uncertainty of the measurement was evaluated to be 1.3% (*k* = 2). In such a case, the relative measurement error between the measurement value and the certified value was calculated to be −0.69%. The relative measurement error slightly increases with the increased concentration, indicating that the PDHID is not suitable for the analysis of SF_6_ with a concentration >100 μmol/mol, when high accuracy is required.

### 2.3. Determination of SF_6_ Using a TCD

**Figure 6:**
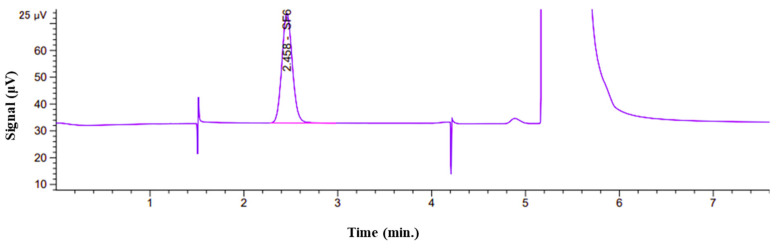
The chromatogram of an SF_6_ CRM (100 μmol/mol) using TCD. The peak at 2.458 min and 5.2~6 min corresponds to SF_6_ and N_2_, respectively.

A typical chromatogram of an SF_6_ CRM using a TCD is shown in Figure 6. The main peak at 2.458 min corresponds to SF_6_. For repeatability, the RSD value of the peak area obtained from six injections of 100 μmol/mol was calculated to be 0.60%.

**Figure 7:**
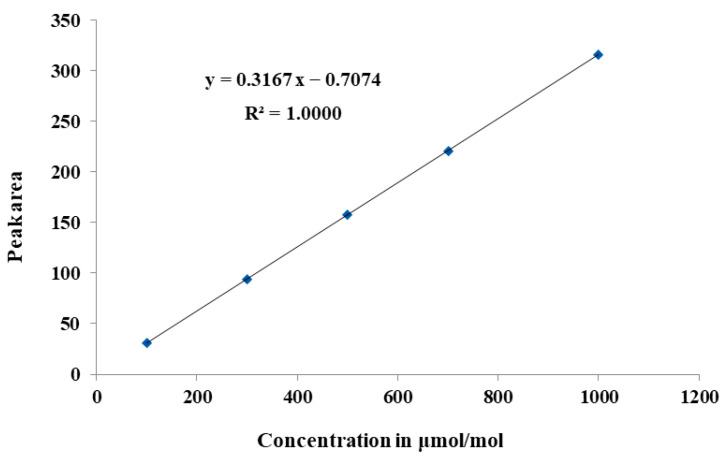
Calibration curves of SF_6_ in nitrogen using GC-TCD, 100~1000 μmol/mol.

A good linearity was obtained in the range of 100–1000 μmol/mol. The linearity coefficient of determination (R^2^) was calculated to be 1.0000, as shown in Figure 7. It indicated that the TCD allows for the higher detection of ranges from 100 μmol/mol to 1000 μmol/mol. To evaluate the accuracy of the calibration curve, an SF_6_ CRM (GBW08124) with a certified value of 100 μmol/mol was chosen as an “unknown” and was analyzed. The measurement value was calculated through using the established equation: *y* = 0.3167 *x* − 0.7074. The measurement value was calculated to be 100.43 μmol/mol. The measurement uncertainty was 1.7% (*k* = 2). In such a case, the relative measurement error between the measurement value and the certified value was calculated to be 0.43%.

### 2.4. Discussion

The detailed characteristics of the relationships between sulfur hexafluoride concentrations and the response from different detectors of gas chromatography are shown in Table 1. An adequate calibration equation is necessary to describe the calibration curves for the SF_6_ analysis. Linear and second polynomial equations were used to verify the adequacy of the equations. The coefficient of determination (*R*^2^) is the sole criterion, and all the numerical values are higher than 0.9995. The relative measurement error was used to evaluate the accuracy of the calibration equation. The results show that the relative measurement error can be decreased when an adequate calibration equation is adopted.

## 3. Experimental

### 3.1. Instruments

The TCD experiments were performed on a gas chromatograph 7890B (Agilent, Santa Clara, CA, USA). A column HayeSep Q (0.91 m, ID 2 mm, mesh size 80/100) was used for GC separation. The sampling volume was set at 1 mL. The sample was injected using a split mode with a split ratio of 10:1. The temperature was set as follows: column oven, 100 °C; inlet, 250 °C; and TCD, 180 °C. Effects of the column oven and TCD temperature on SF_6_ (100 μmol/mol) response are shown in Figures S1 and S2. Helium gas was selected as a carrier gas, and the flow rate was set at 45 mL/min.

GC-PDHID experiments were conducted with a 7890B (Agilent, Santa Clara, CA, USA), which has been custom configured by Wasson-ECE, and includes the columns. This system was designed for the analysis of trace impurities in gas. It is also suitable for the analysis of SF_6_. The temperature was set as follows: column oven, 50 °C, firstly maintained for 8 min, then increased to 100 °C with a gradient of 20 °C/min, maintained for 5.5 min; inlet, 60 °C; and PDHID, 250 °C. Helium gas was chosen as a carrier gas, and the flow rate was set at 30 mL/min.

FPD experiments were performed on gas chromatograph TP-2090E (Tianpu Instrument Co., Guangzhou, China). A column GDX-502 (3 mm ID × 4 m, inert and porous solid particles which are suitable for SF_6_ analysis) was used for GC separation. The temperature was set as follows: column oven, 60 °C; inlet, 60 °C; and FPD, 140 °C. The gas flow rate for the FPD was set as follows: nitrogen, 30 mL/min; hydrogen, 140 mL/min; air, 80 mL/min and 168 mL/min.

### 3.2. Materials

Sulfur hexafluoride in nitrogen CRMs (10.0 ± 0.1 μmol/mol, 100 ± 1 μmol/mol, 0.100 ± 0.001% mol/mol, 0.601 ± 0.006% mol/mol, *k* = 2, GBW08124) were obtained from the National Institute of Metrology (NIM, Beijing, China). The impurities in sulfur hexafluoride and nitrogen raw materials were qualified and quantified when the CRM was being developed according to ISO 6142-1:2015 [25].

Pure nitrogen gas (99.9999%) was provided by Air Liquide (Tianjin, China).

### 3.3. Methods

Repeatability was used to assess measurement precision. The relative standard deviation (RSD) affects the uncertainty of the measurement results. The results were expressed in terms of RSD for the peak areas of SF_6_. Six consecutive injections of SF_6_ CRMs were used to calculate the repeatability, and then to evaluate the precision of the method.

Calibration curves were obtained using the SF_6_ concentration against the peak area from GC with different detectors. Lower concentrations of SF_6_ in the calibration curve were diluted directly through a high concentration SF_6_ CRM, using mass flow controllers (Alicat Scientific, Tucson, AZ, USA). The mass flow controllers were calibrated by the Institute of Metrology, according to the National Verification Regulation. The expanded measurement uncertainty for mass flow controllers’ calibration was in the range of 0.51~0.54% (*k* = 2). An SF_6_ CRM with a certified value was chosen as an “unknown” to verify the accuracy of the calibration curve made using the diluting method with mass flow controllers.

The measurement uncertainty of SF_6_ was evaluated according to the Guide to the Expression of Uncertainty in Measurement (GUM). On the basis of GUM, the standard measurement uncertainty of SF_6_ was combined with the uncertainty of the SF_6_ CRM, the mass flow controllers’ calibration, and repeatability. The expanded uncertainty was obtained via multiplying the standard uncertainty by a coverage factor (*k* = 2).

## 4. Conclusions

In this paper, GC detectors, including an FPD, a PDHID, and a TCD, analyzing SF_6_ were carefully evaluated. For the FPD, the calibration curves have a relatively narrow range. Second polynomial equations were used, fitting a calibration equation. Both the PDHID and TCD have a good linearity, and coefficients of determination (*R*^2^) are equal to 1.0000 in the concentration range of 10–100 μmol/mol, and 100–1000 μmol/mol, respectively. The relative measurement error indicated that the PDHID is more suitable for analyzing SF_6_ with concentrations lower than 100 μmol/mol, whereas the TCD is more suitable for analyzing SF_6_ with concentrations larger than 100 μmol/mol. In summary, an appropriate gas chromatographic detector for the accurate determination of SF_6_, especially for developing SF_6_ reference materials, was crucial to establish a GC method. The results from this study provide guidance for choosing an appropriate detector for the accurate determination of SF_6_, which would subsequently contribute to the development of SF_6_ reference materials used in greenhouse gases’ control.

## Figures and Tables

**Table 1:** Calibration equations for the sulfur hexafluoride concentration and the peak area for GC with different detectors.

Detector	Concentration Range (μmol/mol)	Fitting Method	Calibration Equation	Coefficient of Determination
FPD	10–20,	Second polynomial equation	*y* = 0.2884*x*^2^ + 20.432*x* − 88.378,	*R*² = 0.9995,
	20–40,		*y* = 0.5154*x*^2^ + 27.32*x* − 336.7,	*R*² = 0.9998,
	40–70,		*y* = 0.035*x*^2^ + 3.6795*x* − 63.379,	*R*² = 0.9997,
	70–100,		*y* = 0.0278*x*^2^ + 5.8495*x* − 178.7,	*R*² = 0.9996,
	100–1000,		*y* = −0.0013*x*^2^ + 9.7188*x* − 514.61,	*R*² = 0.9998,
	1000–6000		*y* = 1 × 10^−5^*x*^2^ + 0.5634*x* − 31.361	*R*² = 0.9999
PDHID	10–100,	Linear equation	*y* = 491.6591*x* + 268.6090,	*R*² = 1.0000
	100–1000		*y* = 415.8977*x* + 9408.4270	*R*² = 0.9997
TCD	100–1000	Linear equation	*y* = 0.3167*x* − 0.7074	*R*² = 1.0000

## Data Availability

All the data are available upon reasonable requirement.

## Supplementary Materials

The following supporting information can be downloaded at: https://www.mdpi.com/article/10.3390/molecules29040787/s1, Figure S1. Effect of TCD temperature on SF_6_ (100 μmol/mol) response. Figure S2. Effect of column oven temperature on SF_6_ (100 μmol/mol) response.
