# Supplementary material for: Characteristic Evaluation of Gas Chromatography with Different Detectors for Accurate Determination of Sulfur Hexafluoride
Source: Molecules. 2024 Feb 8;29(4):787. doi: 10.3390/molecules29040787 (PMC10893484; doi:10.3390/molecules29040787)
Supplement: Supplementary file 1 [file molecules-29-00787-s001.zip › molecules-2829431-supplementary.pdf]

Supplementary Information

Characteristics Evaluation of Gas Chromatography with Different Detectors for Accurate Determination of Sulfur Hexafluoride

Susu Pan [1], Tiqiang Zhang [2], Guocheng Zhang [1*], Zhenqi Yang [1], Duan Feng [1], Zhikuan Zhou [1] and Xuelei Ning [1]

[1] Division of Ecology Environment and Energy Resources, Beijing Institute of Metrology, Beijing 100012, China; panss@bjjl.cn (S.P.); yangzq@bjjl.cn (Z.Y.); fengduan@bjjl.cn (D.F.); zhouzq@bjjl.cn (Z.Z.); ningxl@bjjl.cn (X.N.)

[2] Center for Environmental Metrology, National Institute of Metrology, Beijing 100029, China; zhangtq@nim.ac.cn

* Correspondence: zhanggc@bjjl.cn; Tel.: +86-1057176882

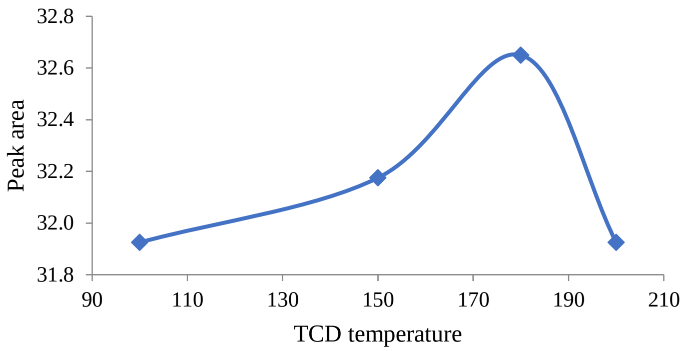

Figure S1. Effect of TCD temperature on SF_6 (100 µmol/mol) response

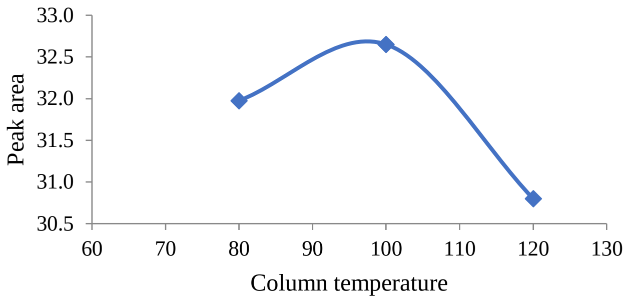

Figure S2. Effect of column oven temperature on SF_6 (100 µmol/mol) response
